# Case Report: Cerebral folate deficiency caused by *FOLR1* variant

**DOI:** 10.3389/fped.2024.1434209

**Published:** 2024-09-12

**Authors:** Qian Wang, Jie Yang, Chunmei Yu, Yao Deng, Qianhui Wen, Hua Yang, Hao Liu, Rong Luo

**Affiliations:** ^1^ Department of Pediatric Neurology, West China Second University Hospital, Sichuan University, Chengdu, Sichuan, China; ^2^ Key Laboratory of Birth Defects and Related Diseases of Women and Children of Ministry of Education, Sichuan University, Chengdu, Sichuan, China

**Keywords:** cerebral folate deficiency, *FOLR1* gene variant, 5-MTHF, developmental delay, seizures

## Abstract

**Background:**

Cerebral folate transport deficiency (CFD) is a rare neurological disease characterized by a deficiency in 5-methyltetrahydrofolate (5-MTHF) in the cerebrospinal fluid, with a normal peripheral total folate level. Late infantile-onset refractory seizures, ataxia, movement disorders, hypotonia, developmental delays, and developmental regression characterize CFD. Some patients present with visual and hearing impairments and autism-like manifestations. This study aimed to elucidate the clinical features, diagnostic approach, and therapeutic outcomes in siblings with CFD due to *FOLR1* variants, highlighting the importance of early diagnosis and treatment.

**Case presentation:**

We reported the cases of two siblings with CFD caused by a new variant in *FOLR1*. They presented with intractable epilepsy, developmental regression, and ataxia, and the younger sibling developed autism. Whole-exon sequencing revealed a c.148G>A homozygous variant, resulting in a change in the amino acid sequence (p.Glu50Lys). Low 5-MTHF levels were detected in the cerebrospinal fluid.

**Conclusions:**

This report illustrates that CFD was caused by *FOLR1* variants in two siblings. They had intractable epilepsy, developmental regression, and ataxia, and a diagnosis of CFD was confirmed by a c.148G>A (p.Glu50Lys) variant in *FOLR1*, a new pathogenic variant in *FOLR1*. Early diagnosis is essential and can improve outcomes in affected patients.

## 1 Background

Cerebral folate transport deficiency (CFD) is a rare autosomal recessive genetic disease caused by a deficiency of 5-methyltetrahydrofolate (5-MTHF) in the cerebrospinal fluid (CSF), leading to various neuropsychiatric symptoms. *FOLR1* is located on chromosome 11q13.4 and contains seven exons. Variants in *FOLR1*, encoding folate receptor alpha (FRα), can cause serious clinical manifestations and brain-specific folate transport deficiency, leading to neurodegeneration in early childhood (1–4). The prevalence of this autosomal recessive disorder is <1 in one million individuals worldwide. FRα is a folate transport system in the central nervous system. The main causes of changes in FRα function include *FOLR1* variants and FRα autoantibodies. *FOLR1-*related cerebral folate transport deficiency (*FOLR1*-CFTD) presents in the second and third years of life. The main clinical manifestations in children with *FOLR1*-CFTD are psychomotor retardation, severe developmental regression, hypotonia, involuntary movement, ataxia, and seizures, some of which are complicated by visual and hearing impairments and autism (3). Brain magnetic resonance imaging (MRI) shows delayed myelination and cerebellar atrophy, magnetic resonance spectroscopy (MRS) shows a decreased choline peak, and electroencephalography (EEG) shows moderate background and multifocal epileptic waves. The 5-MTHF level in the CSF is low (5). This study reports the cases of two siblings with severe clinical manifestations of *FOLR1*-CFTD. The objective of this study in presenting these cases was to highlight that *FOLR1*-CFTD deserves attention and early recognition, and aggressive treatment can improve patient prognosis.

## 2 Case presentation

Two children were born to Chinese parents who were not first- or second-degree relatives. Both patients had an unremarkable family history.

### 2.1 Patient 1

**Figure 1:**
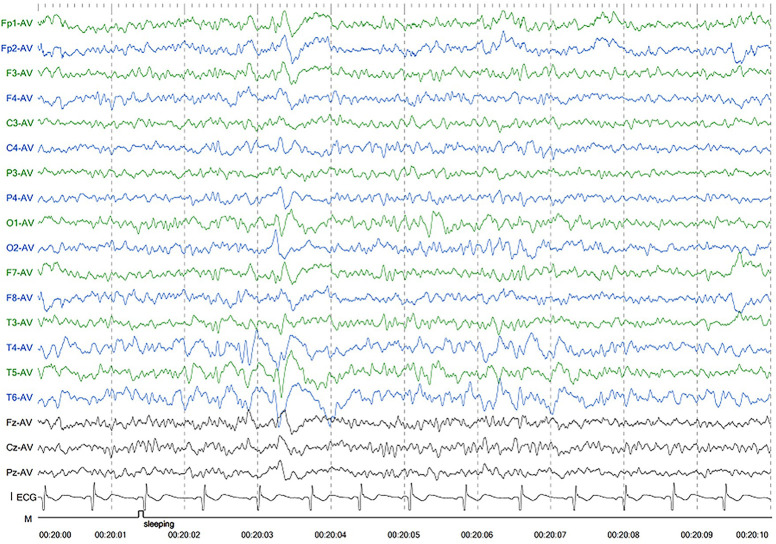
Electroencephalography of patient 1 showed generalized slowing and irregular epileptic discharges.

**Figure 2:**
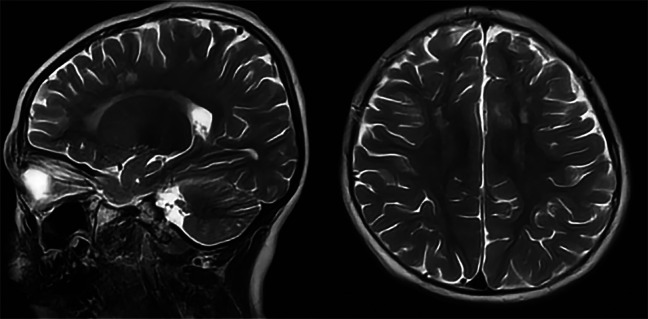
Axial and sagittal T2-weighted MRI of patient 1 showed diffuse high signal intensity within the white matter of the bilateral cerebral hemispheres.

Patient 1 was the first son of healthy Chinese parents, with an uneventful pregnancy, delivery, and neonatal period. His weight, height, and head circumference were within the normal ranges. His motor and mental development were normal up to the age of 3 years. The child experienced evident developmental regression at the age of 3 years. He appeared unsteady on his feet and was prone to falling, followed by ataxia, progressive speech disturbances, decreased gross and fine motor skills, general hypotonia, and apathy. When he was four years old, his parents took him to the hospital for consultation, and the doctor considered delayed development and recommended rehabilitation training; however, rehabilitation showed no significant improvement. When he was 8 years old, he was referred to our hospital, and a neurological examination revealed hypotonia and ataxia. He also experienced myoclonic seizures (several series per day). Oral depakine, an antiepileptic therapy, was ineffective. Long-range EEG showed multifocal epileptiform discharges (Figure 1), with no abnormality in the auditory-evoked potential or visual-evoked examination, suggesting possible visual cortex lesions. Brain MRI showed that the white matter in the bilateral cerebral hemispheres was scattered in abnormal signal foci (Figure 2), these foci presented as hypointense on T1-weighted images and hyperintense on both T2-weighted and fluid-attenuated inversion recovery (FLAIR) sequences, diffusion tensor imaging (DTI) showed discontinuity of white matter fibers in the cingulate tract and corpus callosum bilaterally, and MRS showed the decreased ratio of choline to creatinine in the area of interest.

### 2.2 Patient 2

**Figure 3:**
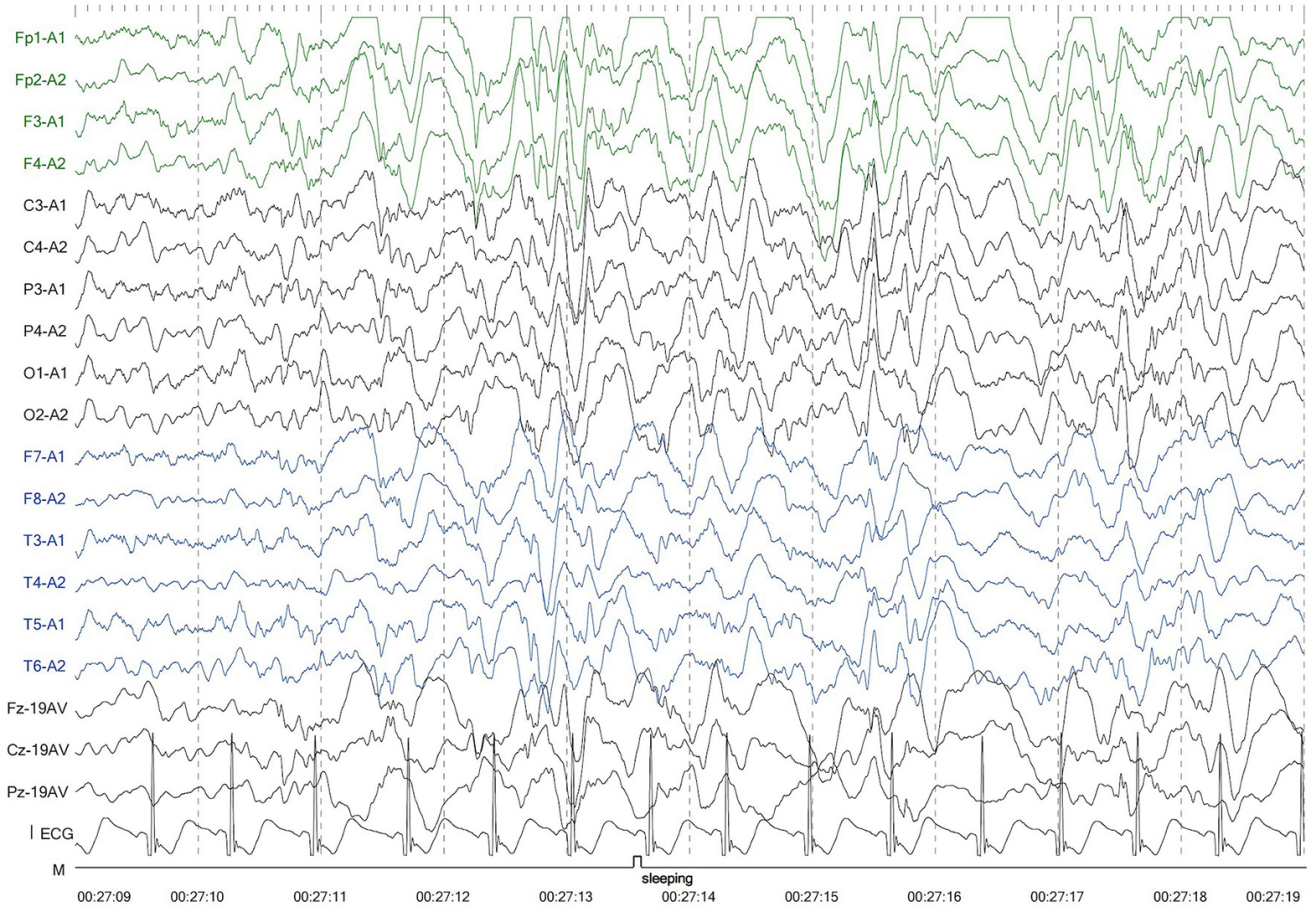
Electroencephalography of patient 2 showed generalized slowing and multifocal epileptic discharges.

**Figure 4:**
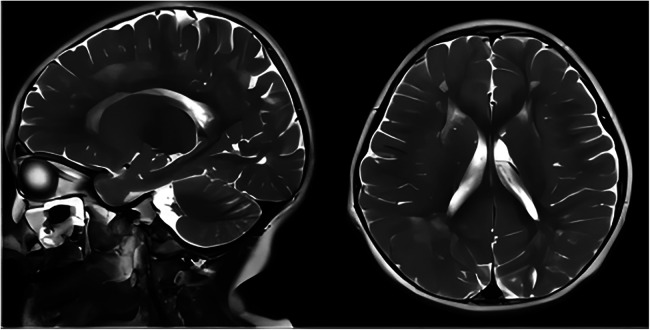
Axial and sagittal T2-weighted MRI of patient 2 showed diffuse high signal intensity within the bilateral white matter of the frontal, parietal, occipital lobes, and periventricular areas. MRI, magnetic resonance imaging.

The second patient was Patient 1's younger sister. The pregnancy, delivery, and neonatal period were uneventful. Her weight, height, and head circumference were within the normal ranges. She experienced major motor developmental regression when she was 3 years old and could not jump forward, with poor balance, poor coordination, and hyperactivity tendencies. The girl gradually showed cognitive regression, reduced language expression, childish behavior, ataxia, and an unsteady gait. When she was 5 years and 8 months old, she was referred to our hospital, and a neurological examination revealed hypotonia and ataxia. She experienced myoclonic seizures (dozens of attacks per day). Her EEG showed abnormalities with a slow background, multifocal epileptiform discharges, and dozens of isolated or cluster seizures (Figure 3). No abnormalities were found in the auditory-evoked potential, and visual-evoked examination suggested possible visual cortex lesions. She was administered oral levetiracetam, deparkin, and topide as antiepileptic treatment; however, her seizures did not improve. Craniocerebral MRI showed abnormal signal shadows in the frontal, parietal, occipital, and ventricular void areas bilaterally (Figure 4), these areas also appeared hypointense on T1-weighted images and hyperintense on both T2-weighted and FLAIR sequences. DTI indicated discontinuity of white matter fibers in the cingulate tract and corpus callosum bilaterally. MRS indicated a decreased ratio of choline to creatinine in the area of interest. The local cerebral sulci in the left parieto-occipital lobe widened, the gyri became thinner, and surrounding glial hyperplasia developed.

Whole-exome sequencing analysis in both patients indicated a c.148G>A homozygous variant, resulting in an amino-acid change (p.Glu50Lys) in FRα (FOLR1). Sanger sequencing confirmed the variant's homozygosity in the affected individuals, with the parents being heterozygous carriers (Supplementary Figure 5).

We suspected that they had CFD. Therefore, we analyzed the 5-MTHF concentration in their CSF (8 and 5 years of age). The CSF analysis revealed low 5-MTHF levels in Patient 1 (23.40 nmol/L; reference values: 60–210 nmol/L) and Patient 2 (11.08 nmol/L; reference values: 70–210 nmol/L).

We conducted scale assessments on the patients using the Wechsler Intelligence Scale for Children, Functional Independence Measure for Children (WeeFIM), and Autism Assessment for Children. The siblings scored <40 points on the Wechsler Intelligence Scale. WeeFIM showed that Patient 1 was mildly dependent on others; however, Patient 2 was heavily dependent on others. The Autism Assessment for Children showed that Patient 1 did not have autism, and Patient 2 had queried autistic symptoms.

Based on clinical symptoms, laboratory tests, and genetic analysis, a FOLR1 variant resulting in decreased 5-MTHF levels in the CSF was determined to be the main cause of the symptoms. When the two siblings were diagnosed with *FOLR1*-CFTD, we immediately initiated treatment with calcium levofolinate, with the dosage gradually increased from 3 mg/kg/day to 5 mg/kg/day. As a result, the frequency of seizures has decreased, and there has been some improvement in language communication compared to that before treatment, although they are still significantly behind their peers. Owing to the short duration of treatment, a long-term treatment plan and continued observation of its efficacy are necessary.

## 3 Discussion and conclusions

The clinical features of the 42 reported *FOLR1*-CFTD patients are summarized in Supplementary Table 1. In this study, we report two Chinese siblings with *FOLR1*-CFTD. *FOLR1*-CFTD was diagnosed based on clinical symptoms and genetic results; the siblings presented with developmental delays, ataxia, and intractable epilepsy.

*FOLR1*-CFTD is genetically heterogeneous. FOLR1 codes for FRα, a membrane protein that is expressed in various cell types. FOLR1 variants can decrease folate levels in the CSF, severely affecting nervous system function. FOLR1 defect was first reported by Steinfield et al. Since 2009, 42 individuals with FOLR1 variants have been identified. These variants include missense (the most common variant), splice, nonsense, and duplication variants. In these studies, normal development preceded the onset of symptoms, which usually appears between the second and third years of life. Motor retardation is a common initial clinical manifestation, and the main clinical manifestations of movement disorders are developmental stagnation and regression, unsteady gait, and ataxia. Some patients are severely handicapped and wheelchair-bound. Most patients developed developmental regression, mental retardation, and frequent therapy-resistant seizures (myoclonic, partial, tonic, tonic-clonic, spam and myoclonic seizures were predominant), and a few patients had no seizures. Some patients developed behavioral abnormalities, including irritability, hyperactivity, and autistic behavior. Most patients had limb spasms, decreased muscle tone, and tremors. Most patients show delayed myelination of the cerebral white matter and cerebellar atrophy on MRI, MRS show low choline and inositol, a slow background, and multifocal epileptiform activity on EEG. All patients had a FOLR1 variant. CSF 5-MTHF was detected in 42 patients, and they had CSF 5-MTHF values below normal levels. The patients in the present study presented developmental regression in their third year of life, similar to patients in most studies. This symptom-free interval between birth and initial symptoms suggests the existence of an alternative transport pathway independent of FRα. A described hypothesis is related to FRβ expression, which may compensate for the loss of FRα function in the first years of life (6). Some patients experience an acute onset of unilateral strabismus and abnormal eye movement before other clinical symptoms become apparent (7). In our patients, the most significant clinical symptoms were ataxia, decreased gross and fine motor skills, and hypotonia. Daily seizures occurred during the course, partly provoked by light changes, and antiepileptic medications were ineffective. Similar to other patients with *FOLR1*-CFTD, our patients’ EEG revealed slow background activity and multifocal epileptic activity. On the MRI, the siblings did not show pronounced cerebellar atrophy, a common imaging finding in patients with FOLR1 variants, and the genetic test results showed FOLR1 variant, which was consistent with the clinical symptoms.

In addition to primary cerebral folate deficiency, which can decrease the level of 5-MTHF in the CSF, nutritional deficiencies, medication effects, liver diseases, mitochondrial disorders, and certain enzyme deficiencies can lead to secondary decreases in 5-MTHF levels in the CSF. Therefore, further differential diagnosis requires the testing of blood amino acids, carnitine profiles, and urinary organic acids; if necessary, genetic testing can be performed for a definitive diagnosis. *FOLR1*-CFTD is a treatable condition, and previous literature reports have emphasized the importance of early treatment with folinic acid. Most patients experienced symptom relief, and in some cases, complete recovery following timely treatment. According to previous studies and the cases reported in the present study, the age of diagnosis is often significantly later than the age of onset. It is somewhat unusual that after repeated inquiries into the medical history, the family described both siblings as normal before the age of 3, after which they experienced neurological regression. This is later than the typical onset time reported in previous literature; however, there may have been more subtle neurological and developmental findings before the children turned 3 that were simply not noticed, and the delayed diagnosis may have led to delayed medication efficacy.

In conclusion, we report a novel pathogenic variant of FOLR1. The literature review and two cases reported in this study show that the clinical course of CFD due to FOLR1 variants largely overlaps with that of metabolic epileptic encephalopathies. When children present with severe developmental regression, ataxia, intellectual disability, and intractable epilepsy, CFD due to FOLR1 variants should be considered, and a genetic test should be performed immediately after symptom onset. *FOLR1*-CFTD is treatable; however, the clinical course can deteriorate without treatment (8). Folinic acid therapy is efficient in treating clinical symptoms, and the earlier the treatment is administered, the better the response (9). When older siblings are diagnosed, treating younger siblings who are asymptomatic or have mild symptoms may prevent the neurological signs of the disease and lead to a significant or complete regression of the neurological symptoms (10). Early diagnosis and treatment are crucial for preventing irreversible cognitive deficits and can improve cognitive function, seizures, walking ability, and language skills.

## Data Availability

The datasets presented in this study can be found in online repositories. The names of the repository/repositories and accession number(s) can be found in the article/Supplementary Material.

## Supplementary material

Supplementary Table 1 Clinical, imaging and biochemical and genetic data of patients with *FOLR1*-related cerebral folate deficiency. EEG, electroencephalography; F, female; FLAIR, fluid-attenuated inversion recovery; M, male; MRI, magnetic resonance imaging.
