# Supplementary material for: Case Report: Cerebral folate deficiency caused by FOLR1 variant
Source: Front Pediatr. 2024 Sep 12;12:1434209. doi: 10.3389/fped.2024.1434209 (PMC11424398; doi:10.3389/fped.2024.1434209)
Supplement: Supplementary Table 1 [file Table1.pdf]

Patient	Sex	Age at onset	Age of diagnosis	Initial symptoms	Main symptoms	MRI/MRS	EEG	CSF 5-MTHF levels (nmol/L)	Mutations	Reference
1	M	2.5 years	4.5 years	Not stated	Motor dysfunction, developmental regression, severely handicapped, wheelchair-bound, therapy-resistant epileptic seizures	Severely disturbed myelination affecting the periventricular and the subcortical white matter when compared to age-matched controls	Not stated	1.4 (43–159)	c.352C>T; (p.Gln 118*) and c.525C>A; (p.Cys175*)	(2)
2 (sib of 1)	F	2 years 3 months	2.5 years	Motor symptoms	Motor symptoms	Mild cerebellar atrophy only	Not stated	<5 (43–159)	c.352C>T; (p.Gln 118*) and c.525C>A; (p.Cys175*)	(2)
3	F	2 years	5 years	Not stated	Severely handicapped, mentally retarded, frequent epileptic	Hypomyelination, cerebellar atrophy, focal T2-hyperintense	Not stated	<5 (43–159)	c.130_147dup; (p.Lys44_Pro49dup)	(2)

					seizures	white matter lesions				
4	M	2.5 years	5 years	Resting and intention tremor	Ataxia, progressive speech disturbances and loss of motor capabilities, general hypotonia, apathy, autism, and myoclonic-astatic seizures	Delayed cortical myelination	Slowing of background activity, paroxysmal dysrhythmia with high amplitude delta wave, and centrotemporal epileptic potentials bilaterally	Below the detection limit	c.352C>T (p.Q118X) and c.525C>A (p.C175X)	(6)
5 (sib of 4)	F	2.5 years	2.5 years	Not stated	Normal cognitive development, mild truncal ataxia, tremor	Not stated	Mild non-specific slowing of background activity	Below the detection limit	c.352C>T (p.Q118X) and c.525C>A (p.C175X)	(6)
6	F	1 year	Not stated	Motor developmental delay	Myoclonic, tonic seizures, developmental regression, hypotonia, ataxia, athetosis,	Hypomyelination cerebellar and cerebellar atrophy, T2-hyperintense white matter lesions, low choline, normal	Slow background rhythm, multifocal spikes, and sharp and slow waves	5	p.C169Y, p.C169Y	(3)

					hyperreflexia, Babinski sign, distal spasticity	myoinositol				
7	M	1 year 10 months	Not stated	Seizure, developmental delay	Myoclonic SE, developmental regression, hypotonia, ataxia, autistic signs	Delayed myelination, T2-hyperintensity of the periventricular and subcortical white matter, cerebellar, normal choline, and normal inositol	Abnormal background rhythm, multifocal sharp and slow sharp waves	<5	p.C65W, p.C65W	(3)
8	F	3 months	15 years	Congenital microcephaly, tonus imbalance	SE (partial, myoclonic, tonic, tonic-clonic), developmental regression, ataxia, hypotonia, hyperreflexia and distal spasticity, aggressive behavior, very little verbal	Hypomyelination cerebellar and cerebral atrophy	Slow background rhythm, spikes, and slow spike-wave discharges	3	p.C169Y, p.C169Y	(3)

					communication					
9	F	3 years	Not stated	Temporarily nystagmus in infancy, 3 years of gait problems	SE (2 status epilepticus, no other seizures), developmental regression, ataxia, tremor, hypotonia, hyperreflexia	Delayed myelination, cerebellar and cerebral atrophy, T2-hyperintensity in corticospinal tracts	Slow background rhythm, frontal discharges during SE	5	p.C169Y, p.C169Y	(3)
10	F	2 years	Not stated	ataxia	SE (myoclonic, few tonic-clonic), developmental regression, ataxia, aggressive behavior	Irregular myelination, cerebellar atrophy, very low choline, low inositol	Slow background rhythm, multifocal spikes, generalized slow spikes, and wave discharges	<5	p.C169Y, p.N222S	(3)
11	M	1.5 years	Not stated	Motor developmental delay	SE (myoclonic, tonic, atonic, and psychomotor seizures), developmental regression,	Hypomyelination T2-hyperintense white matter lesions, cerebellar atrophy	Slow background rhythm, multifocal spikes, and sharp waves	5	p.C169Y, p.C169Y	(3)

					hypotonia, ataxia, athetoid movements, hyporeflexia, distal spasticity					
12	F	2 years	Not stated	Global developmental delay	SE (tonic-clonic, sometimes short myoclonic), developmental regression, ataxia, tremor, slight hypotonia, gait problems, hyperreflexia, Babinski signs on both sides, autistic signs associated with anxiety and little speech,	Hypomyelination cerebellar and cerebral atrophy, thin corpus callosum	Slow background rhythm, multifocal sharp waves	<5	g.3576T>G, g.3576T>G	(3)
13	M	1 year	7 years	Progressive ataxia, gait,	Cognitive impairment and language delay, SE (tonic and	Diffuse abnormal white matter signal in cerebral hemispheres, focal	High amplitude background activity, frequent myoclonic jerks,	2	c.313T>C (p.Cys105Arg)	(7)

				instability, and jerky tremor	myoclonic), choric movements and multiple, involuntary movements, no social smile, lacked language communication, and only produced incomprehensible sounds	areas of T1 hypointensity and T2 hypointensity at the periventricular white matter and centrum semiovale. Spectroscopy in the right parietal subcortical white matter detected reduced choline, mild atrophy of the vermis	and tonic seizures			
14	M	6 months	8 years	Delayed motor development, profound generalized hypotonia with poor	Dysmetric movements of the arms, ataxia, and moderate psychomotor delay, lacked language communication	Progressive demyelination in frontal and parietal lobes, which also extended into the brain stem, reduced choline levels in the parietal white matter and basal ganglia	Continuous irregular high voltage 1–3 Hz multifocal sharp wave activity in a “hypsarrhythmia-like” pattern	Below the detection limit	p.R204X, p.R204X	(8)

				head control and dysphagia						
15	M	2.5 years	5 years	Hyperactive behavior	Generalized ataxia and intellectual disability, generalized choric movements, SE (myoclonia), irritability, limited use of language, and progressive microcephaly	Generalized hypomyelination, cerebellar atrophy, reduced peaks of myoinositol and choline within the parietal white matter and basal ganglia, bilateral calcifications within the basal ganglia	Impressive deterioration with high-voltage spike and sharp wave activity and a slow high-amplitude background activity	Below the detection limit	p.R204X, p.R204X	(9)
16	M	9 months	5 years 8 months	Delayed motor development	SE (drop attacks and myoclonic jerks), global developmental delay, autistic spectrum disorder,	Subcortical white matter with abnormal high intensity consistent with hypomyelination with cerebellar	Generalized slowing and multifocal epileptic discharges, with occasional attenuation of	7 (40–128)	c.398C>A (p.Pro133His)	(10)

					generalized hypertonia, hyperreflexia, downward planters with an unsteady wide-based gait	atrophy	EEG activity			
17 (sib of 16)	F	4 year s	4 years	SE	SE (drop attacks), global developmental delay	Increased signal intensity in subcortical white matter, putamen, and head of caudate nucleus, and sub-insular and anterior limbs of internal capsule hypomyelination with cerebellar atrophy	Generalized slowing and multifocal epileptic discharges, with occasional attenuation of EEG activity	11 (40–150)	c.398C>A (p.Pro133His)	(10)
18	M	1 year	17 years	Ataxic gait	Ataxia, intention tremor, slurred speech, developmental delay, intellectual	Cerebellar atrophy, brainstem atrophy, cerebral white matter atrophy calcification at subcortical white	Not stated	Below the detection limit	c.374G>T (p.R125L), c.466T>G (p.W156G)	(11)

					disability, mild hypotonia, spastic lower limbs, pyramidal sign, epileptic seizure	matter				
19 (sib of 18)	F	2 years	14 years	Ataxic gait	Ataxia, intention tremor, slurred speech, developmental delay, intellectual disability, mild hypotonia, pyramidal sign, epileptic seizure	Cerebellar atrophy, calcification at basal ganglia and subcortical white matter	Not stated	5	c.374G>T (p.R125L), c.466T>G (p.W156G)	(11)
20	F	9 months	5 years	Persistent gross motor difficulties with problems running and climbin	Developmental delay, challenging behavior and rarely smile, little interest in other people, irritable, exhibiting poor eye contact and several stereotypies,	High signals in the white matter in T1, T2, and FLAIR sequences, indicative of diffuse hypomyelination; depletion of white matter choline on spectroscopy	Low background rhythm and multifocal epileptiform discharges	<1	c.332G>T (p.Glu108X), c.373G>T (p.Arg125Cys)	(12)

				g stairs	ataxic gait, weak deep-tendon reflexes, mild psychomotor delay, SE (myoclonic jerks), absence of language, severe axial hypotonia, choric movements rendering the child wheelchair-bound					
21 (sib of 20)	F	9 months	3 years 1 month	Motor delay	Motor delay, arrested language development, SE (myoclonic and tonic)	Similar to 21	Multifocal epileptiform discharges	<1	c.332G>T (p.Glu108X), c.373G>T (p.Arg125Cys)	(12)
22	F	2 years	36 years	Not stated	SE	Not stated	Not stated	<10	c.128A>G (p.His43Arg)	(13)
23	F	1 year	33	SE (myocl	Ataxia, developmental	Not stated	Not stated	<10	c.128A>G	(14)

		10 months	years	onic)	regression, lack of language, SE (myoclonic, tonic-clonic)				(p.H43R)	
24 (sib Of 23)	F	1.5 years	28 years	Progressive ataxia and developmental regression	Developmental regression, feeding difficulties, only a few words of speech, wheelchair-dependent, SE (myoclonic, tonic-clonic)	Extensive periventricular and deep cerebral white matter changes with frontal lobe and cerebellar atrophy	Not stated	<10	c.128A>G (p.H43R)	(14)
25	F	1.5 years	7 years	SE (unconscious episode)	Mental regression, cognitive and physical skills deteriorated with progressive ataxia, hypotonia with mild spasticity, myoclonic, SE	Symmetrical white matter hyperintensity on T2-weighted and FLAIR images, cortical laminar necrosis and ulegyria, cerebellar atrophy, reduced choline peak	Diffuse high amplitude slow waves intermingled with sharp waves or spikes, with sleep EEG, diffuse poly spikes and slow waves were	0.5	c.466T>G (p.W156G)	(15)

					(tonic)		frequently evident			
26	F	3 years	9.5 years	Tremors and drooling	Speech delay and poor cognitive abilities, no SE, moderate to severe mental retardation	Non-specific, diffuse T2 hyperintensity of white matter in both cerebral hemispheres in subcortical, centrum semiovale and deep periventricular regions, more prominent posteriorly	Slow background activity of 3–5 Hz, no asymmetry between the two hemispheres, infrequent high amplitude polymorphic sharp and slow waves with and without spikes over the frontocentral head regions	Not stated	c.665A>G (p.Asn222Ser)	(16)
27	F	1.5 years	3 years	Ataxia, tremor, irritability, sleep disorders, and speech difficult	SE (myoclonic), dyskinesia, cerebral atrophy, and the patient's speech abilities and motor function had gradually	Severe hypomyelination	Slow activity and a spike-and-wave pattern associated with an epileptiform activity	1	c.197G>A (p.Cys66Tyr)	(17)

				ies	declined, and her independent walking ability was lost					
28	M	1.5 years	6 years 11 months	Mental delay and mental retardation	Global developmental delay, slurred speech, hyperactivity, sialorrhea, difficulty in managing behavior, unsteady gait, hypotonia	Encephalomalacia and laminar necrosis in the brain left parietotemporal lobe, hippocampus, and bilateral frontal lobe with diffuse white matter disorder	Not stated	Below the detection limit	c.524G>T (p.C175F)	(18)
29	F	12months	2 years	Developmental delay, hypotonia and mild ataxia	Important developmental regression, ataxia, autistic behavior and seizures (infantile spasms and other)	Delayed myelination, low Cho and Ino	Abnormal EEG background	19	c.195C>G(p.C65W), c.427T>A(p.W143R)	(21)

30(sib Of 29)	M	12months	12months	Developmental stagnation and delay, dyskinesia	Hypotonia, dyskinesia	Delayed myelination, low Cho and Ino	Normal	49	c.195C>G(p.C65W), c.427T>A(p.W143R)	(21)
31	M	1.5 years	8 years	Gait ataxia, speech delay	Cerebellar, bulbar, pyramidal	Supratentorial hypomyelination, affected corpus callosum, cerebellar hypomyelination and atrophy, low Cho	Slow background activity and multifocal epileptiform discharges	Not stated	c.465_466delinsTG (p.W156G)	(22)
32(sib Of 31)	F	22 months	2 years	Intention tremor in upper limbs	Intention tremor in upper limbs, truncal ataxia	Cerebral and cerebellar hypomyelination, thin corpus callosum, low Cho	Not stated	Not stated	c.465_466delinsTG (p.W156G)	(22)
33	M	3 year	11 years	Speech delay	Tonic clonic seizure, lost motor skills and	Ventricular system dilatation and abnormal white	Not stated	<10	c.245A>G (p.Tyr82Cys)	(23)

		s			language abilities, spastic quadriplegia	matter signals				
34(sib Of 33)	F	5 years	13 years	Seizures(absence, tonic-clonic)	Lost motor and speech abilities, spastic quadriplegia	Thinning of the corpus callosum, dilatation of the ventricular system and cerebellar atrophy	Not stated	<10	c.245A>G (p.Tyr82Cys)	(23)
35(sib Of 33)	F	9 months	18 years	Global delay	Developmental regression, irritable, nonverbal, unable to stand independently, ataxia, SE(tonic-myoclonic)	Diffuse white matter signal abnormality and cerebellar atrophy, low levels of choline and myoinositol in the white matter	Activity low-voltage background and multifocal epileptiform discharges	Not stated	c.428G>A (p.Ty143*)	(24)
36(sib Of 35)	F	10 months	16 years	Strabismus in the left eye	Psychomotor regression, SE(tonic, myoclonic,	High signal abnormality in the fronto-parietal white matter, cerebellar atrophy, reduced	Multifocal EEG abnormalities	Not stated	c.428G>A (p.Ty143*)	(24)

					absences seizures)	choline and myoinositol				
37(sib Of 35)	M	9 mont hs	15 years	Right eye esotropi a	Behavioral and developmental regression, loss of acquired speech, ataxia, SE(atonic, tonic and myoclonic)	Scattered signal abnormalities in the frontal and parietal white matter, cerebellar vermis hypoplasia	High-amplitude slow background activity	9	c.428G>A (p.Ty143*)	(24)
38	F	18 moth s	6years	Autistic spectru m disorde r	SE(myoclonic-ato nic), mental regression, ataxia, hypotonia and dysmetria	Cortical atrophy	Slowing of background activity and generalized spike/polyspike-w ave discharges with high amplitude	<1.6	c.665A>G(p.A sn222Ser)	(25)
39	F	3 year s	21 years	Slight difficult ies in speech and fine	Psychomotor regression, dystonia, progressive gait difficulties, cognitive	Bilateral T1 hyperintensities in the temporo-parietal, periventricular regions and basal	Diffuse encephalopathy as well as generalized slow spike wave complexes and	<2	c.383G>A;(p.R 128Q)	(26)

				motor skills	impairment and obesity	ganglia	multifocal spikes			
40	M	47 years	47 years	Double vision and postural instability	Double vision, postural instability, ataxia and vertical diplopia in the left/upper left gaze position	Diffuse, bilateral, and symmetric supratentorial hyperintensity on FLAIR images	Not stated	10	c.45G>T and c.493+2T>C	(27)
41	M	3.5 years	33 years	SE	SE(absence, myoclonic, tonic-clonic), cognitive dysfunction, dysarthria, quadriparesis	Cerebral and cerebellar atrophy, calcification in the bilateral globus pallidus, and cerebellum	Diffuse background slowing and sleep-activated generalized epileptiform anomalies being prominent in left frontotemporal regions	0.6	c.610C>T,(p.Arg204Ter)	(28)
42(sib Of 41)	M	2 years	23 years	SE	SE(tonic, tonic-clonic, absences), cognitive	Hypomyelinated area in the left parietooccipital region, cerebellar	Diffuse background slowing, prominent	10	c.610C>T,(p.Arg204Ter)	(28)

					dysfunction, dysarthria, quadriparesis, bilateral internal gaze palsies	atrophy, and calcification in globus pallidus bilaterally	generalized epileptiform discharges			
43	M	3 years	8 years	developmental regression	ataxia, progressive speech disturbances, decreased athletic ability, general hypotonia, apathy and SE	ataxia, progressive speech disturbances, decreased athletic ability, general hypotonia, and apathy,ataxia, progressive speech disturbances, decreased athletic ability, general hypotonia, and apathy.	multifocal epileptiform discharges	23.4	c.148G>A;(p.Glu50Lys)	Our patient/1
44	F	3 years	5 years 8 months	motor developmental regression	cognitive regression, reduced language expression, childish behavior, ataxia, unsteady	abnormal signal shadows in the frontal, parietal, occipital, and ventricular void areas bilaterally, choline to creatinine	slow background, multifocal epileptiform discharges, and dozens of isolated or cluster seizures	11.08	c.148G>A;(p.Glu50Lys)	Our patient/2

					gait, SE	in the area of interest				
